# 3DFlu: database of sequence and structural variability of the influenza hemagglutinin at population scale

**DOI:** 10.1093/database/baw130

**Published:** 2016-10-01

**Authors:** Giovanni Mazzocco, Michal Lazniewski, Piotr Migdał, Teresa Szczepińska, Jan P. Radomski, Dariusz Plewczynski

**Affiliations:** 1 Centre of New Technologies, University of Warsaw, Warsaw, Poland; 2 Institute of Computer Science, Polish Academy of Sciences, Warsaw, Poland; 3 Department of Physical Chemistry, Faculty of Pharmacy, Medical University of Warsaw, Warsaw, Poland; 4 Interdisciplinary Centre for Mathematical and Computational Modeling, University of Warsaw, Warsaw, Poland; 5 Faculty of Pharmacy, Medical University of Warsaw, Warsaw, Poland

## Abstract

The influenza virus type A (IVA) is an important pathogen which is able to cause annual epidemics and even pandemics. This fact is the consequence of the antigenic shifts and drifts capabilities of IVA, caused by the high mutation rate and the reassortment capabilities of the virus. The hemagglutinin (HA) protein constitutes the main IVA antigen and has a crucial role in the infection mechanism, being responsible for the recognition of host-specific sialic acid derivatives. Despite the relative abundance of HA sequence and serological studies, comparative structure-based analysis of HA are less investigated. The 3DFlu database contains well annotated HA representatives: 1192 models and 263 crystallographic structures. The relations between these proteins are defined using different metrics and are visualized as a network in the provided web interface. Moreover structural and sequence comparison of the proteins can be explored. Metadata information (e.g. protein identifier, IVA strain, year and location of infection) can enhance the exploration of the presented data. With our database researchers gain a useful tool for the exploration of high quality HA models, viewing and comparing changes in the HA viral subtypes at several information levels (sequence, structure, ESP). The complete and integrated view of those relations might be useful to determine the efficiency of transmission, pathogenicity and for the investigation of evolutionary tendencies of the influenza virus.

**Database URL**: http://nucleus3d.cent.uw.edu.pl/influenza

## Introduction

The influenza virus type A (IVA) is one of the most important, untamed pathogens, which can easily overcome the immunological control of a host and escape the pharmaceutical treatment with specific drugs (1). The virus is responsible for recurrent epidemics causing hundreds of thousands of fatal cases annually (2). The hemagglutinin (HA) protein is the main antigen of IVA and exhibits a remarkably high degree of polymorphism. The HA regulates the influenza virus entry into the host cell, thus it has a strong impact on both pathogenicity and epidemiological consequences of the IVA infection. Furthermore the protein plays a crucial role in the virus’ mechanism of action, defining the likelihood of the interaction between the host cells (e.g. human, swine, chicken, mallard, duck and other bird and mammal species) and the infecting virus. The specific sequence and structural features of the binding site of a given HA protein are responsible for the differential recognition of host-specific sialic acid derivatives (3, 4). Hence the analysis of HA variability at the molecular level is crucial for the correct understanding of the specific organisms’ risk of infection. For example, it has been recently shown that some strains of A/H5N1 serotype, isolated from birds, can convert to human-specific strains. These strains can effectively infect mammals by airborne droplet transmission, by mutating only four positions in its HA (5). Although the analysis of HA genetic variability is classically applied to sequence and serological data, a comparative analysis of HA structure-based data could enhance the prediction of potentially dangerous viral strains, allowing the estimation of risk of pandemics.

The 3DFlu database contains a collection of HA homology models, which represent the receptor binding site variability of this protein across the most common influenza strains. Only specific IVA-serotypes (H1, H2, H3, H5, H7, H9, H10) were chosen as they represent either serotypes which caused human pandemics in the last century: 1918 (H1), 1957(H2) and 1968 (H3) (6) or the avian-infecting serotypes (H5, H7, H9, H10) for which sporadic human infections with high fatality rate were recorded (7–10). Another criterion for selecting this specific set of serotypes was the availability of multiple crystallographic structures. Additionally, HA structures obtained from the Protein Data Bank (PDB) database (11) were also included. Insightful structure-based information for all the entries are provided, along with the metadata obtained from different public sources. The web-application was designed to enable the easy access to the resources available in the database. These include the visualization of structures, sequences and various types of relations between the proteins.

Several influenza-focused web-accessible databases such as: the NCBI Influenza Virus Resource (http://www.ncbi.nlm.nih.gov/genomes/FLU/FLU.html (12), Global Initiative on Sharing Avian Influenza Data EpiFlu Database (GIS-AID, http://www.gisaid.org) (13), the Influenza Virus Database, (http://influenza.psych.ac.cn/) (14), the OpenFlu Database (http://openflu.vital-it.ch) (15) and the Influenza Research Database (IRD, http://www.fludb.org) (16), are already available. However 3DFlu presents a unique aggregation of HA-selected information. The interactive exploration of these proteins and their relations is facilitated by the provided web interface. With our database researchers gain a tool to access several layers of information about influenza virus HA. Such integrated view might be useful to determine the efficiency of transmission, pathogenicity and evolutionary tendencies of the virus. The collected models can be utilized by other researchers in applications such as virtual high-throughput screening for designing new HA inhibitors, or prediction of a host shift, by a given IVA serotype that might results in a global epidemic.

## Methods

### Data sources

The HA sequences were extracted from the following databases: Uniprot (http://www.uniprot.org/), NCBI (http://www.ncbi.nlm.nih.gov/), EMBL-EBI (http://www.ebi.ac.uk/), IRD (https://www.fludb.org/) and GISAID (http://platform.gisaid.org/). The metadata were retrieved from the original data sources and afterwards manually curated. For each entry the following metadata were retrieved: virus subtype, year of the infection, host species and geographic location of the infected host. It must be noted that in four cases (ABD79101, ABD79112, AGQ47740, AGQ47776) information about localization is missing while for three entries (AGO00313, AGO00337, AGQ47835) no date of infection is available. All the available crystal structures of HA were downloaded from the PDB (http://www.rcsb.org). The structures were selected on the basis of their completeness (HA fragments were removed) and crystallographic quality. Structures with resolution values >3.0 Å or free *R*-values > 0.3 were automatically discarded.

### Homology modeling

The templates for the homology modeling were selected using BLASTp (version 2.2.28+) (17). For each target sequence the closest available structure, having a resolution value lower than 3.0 Å and a free *R*-value lower then 0.3, was selected as a template. Multiple sequence alignments (MSAs) between templates and target sequences were constructed using the PCMA software (version 2.0) (18). The models were built using MODELLER (version 9.15) (19). Ten structures were reckoned for each model. For each entry the pseudo-energy criterion, returned by the method, was used to select the best model. The quality of each model was further assessed using the Qmean (20) and Molprobity (21) servers. Both servers provide access to scoring functions which estimate the quality of the models, also allowing the identification of potentially unreliable regions. The validation of our homology modeling routine is provided in the Supplementary Materials.

### Structural analysis

TM-align (22) was used for the structural alignment of PDB structures and protein models. The Adaptive Poisson-Boltzmann Solver software package (APBS, version 1.4.1) (23) was then applied to compute the electrostatic potentials (ESP). The center of the HA’s globular domain was empirically selected as the center of the ESP grid. The grid dimensions were 56.0, 44.8 and 44.8 Å, while the number of points per axis were 161, 129 and 129 for the x, y and z axes, respectively. The distance between each point in the cubic grid is therefore 0.35 Å.

Similarities between protein pairs were measured in terms of sequences, structures and electrostatic potentials. The sequence similarity score was obtained applying the Python library pairwise2 (http://biopython.org/). The proposed global sequence alignment was scored using BLOSUM62 matrix. The Root-mean-square deviations (RMSD) of atomic positions were computed using TM-align (22). Finally the ESP similarities were obtained from the Protein Interaction Property Similarity Analysis software (24). The MSA of the protein sequences was performed using PCMA (18) and 3D-Coffee (25). The secondary structural elements for each model were computed with the use of DSSP (26). The mobility of protein residues was estimated applying the Gaussian Network Model (GNM). The Python library ProDY (27) containing the GNM implementation was used for this purpose. Selected amino acid indexes were applied to the protein sequences in order to describe them in terms of specific physico-chemical properties. The following list of High-Quality Indexes (28) was extracted from the Amino Acid Index Database (29): BLAM930101, BIOV880101, MAXF760101, TSAJ990101, NAKH920108, CEDJ9 70104, LIFS790101, MIYS990104. The schematic representation of the data preparation is given in the Supplementary Figure S1. If not stated otherwise, the default parameters were used for all the above mentioned programs.

### Protein network visualization

Each protein is defined as a node in a force-network model. Each edge has an elastic constant defined as a function of the distance between the two proteins considered, using the selected metric. The spring constant *k*_ij_ is defined by the Equation 1, where the distance scale *d* and the exponent *a* are chosen empirically. Moreover, for performance reasons only the links having a value above a manually set threshold were included in the computation. The position was computed applying the force layout from the D3.js library (https://d3js.org/).

(1) $$d_{ij}=e^{-{\left(\frac{d_{ij}}{d}\right)}^{a}}$$

### Database structure

The database is available for download as a single compressed file. It contains the following data:

- Metadata for all the entries.
- Processed 3D structures for both PDB entries and models.
- Distance matrices (RMSD, ESP, sequence similarity).
- Sequence and structural alignments.
- HQI indexes.
- Electrostatic potential grids calculated with APBS.

The data structure is described in details in the Supplementary Materials.

## Results

### Data

Initially all the available sequences of the H1, H2, H3, H5, H7, H9, H10 serotypes were extracted from publicly available databases. This procedure resulted in 22 800 unique sequences. According to our estimations up to 7% of those might contain different types of errors. In order to enhance the quality of the final data, the original data were initially manually processed. The sequences containing ambiguous IUPAC codes (http://www.bioinformatics.org/sms/iupac.html) were treated as uncertain and consequently excluded. From the obtained set only unique sequences were selected. If multiple sequences shared the same gene identifier the one with the earliest date of sample isolation was chosen as a representative of that subset. As a consequence out of the 22 800 sequences originally downloaded 21 984 were maintained in the curated version of the sequence database. For modeling purposes a subset of sequences was selected. Firstly the amino acids responsible for the interaction with sialic acid derivatives were selected. These include K133A, G134, V135, T136, A137, T155, K156, P185, P186, D190, S193, L194, D225, Q226, A227, G228 (amino acids and their numbering as in 1RD8 structure, representing the earliest recovered, full length HA sequence of the Spanish flu 1918–1920). Secondly all the sequences sharing identical residues at the above mentioned positions were grouped. From each group a single sequence was selected. In our opinion the resulting 1192 proteins are sufficient to describe the sequence variability of the HAs receptor-binding site. The workflow of the data preparation is described in the Supplementary Figure S1.

The HA glycoprotein is a trimer, with each monomer synthesized as a single polypeptide chain (HA0). Next HA0 is cleaved into two subunits, HA1 and HA2. The former contains globular region encompassing the receptor binding site and regions recognized by host immune system. The latter is folded mainly into a helical coiled-coil structure and is responsible for both the fusion of the host and the virus membranes (30) and the attachment of HA to the virus lipid bilayer. In our database, the HA2 subunit was removed from each analysed sequence due to its high sequence conservation. Furthermore all the sequences were aligned and the flanking amino acids not present in all sequences were discarded. This allowed the preparation of sequences with similar length. Each of such pre-processed sequence was then used as a query for template identification. The template with highest percentage of identity to the query sequence and highest coverage value was selected for modeling. This procedure resulted in 43 templates. Next all entries were superimposed over one of the structures in the database (PDB id: 4EDA) for the visualization purpose.

The obtained proteins originate from IVA infecting two different classes of organisms (aves and mammalian) and over 250 different species. The most represented species are pig (*Sus scrofa*, 485 sequences) and human (*Homo sapiens*, 381 sequences). Avian are collectively well represented (324 sequences). The hosts’ infection occurred between 1918 and 2014 in six different continents. Moreover, seven different subtypes are present in our database. Detailed information is available in the Supplementary Figure S3.

Out of 286 proteins meeting the initial filters, a collection of 263 PDB structures passed the validation procedure described in Methods. Just as for models the HA2 subdomain and the N- and C-terminus amino acids of HA1 were removed from PDB structures.

In contrast with the balanced host distribution found within the models, human IVAs are overrepresented among the crystallographic structures (171) (Supplementary Figure S4). Moreover, the majority of crystallographic structures originate from Asia (145).

Our database focuses mainly on the structural variability of the HA1 globular region containing the receptor binding site. In the HA crystal structures, the spatial orientation of the globular region with respect to the long helical one can vary significantly from one protein to another. Thus the simultaneous superimposition of two complete structures might result in the incorrect alignment of their respective globular regions. As a result the superposition was performed considering only the HA globular region. This procedure allowed us to compare proteins in terms of structural-based properties. The RMSD matrix of the superimposed entries was initially computed with TMalign and further used for the visualization (as described in the ‘Web interface’ section). As expected, the proteins having identical HA serotype (e.g. H1) are generally clustered together. However some serotype groups intersect (e.g. H1 and H9) suggesting a possible evolutionary relation. Interestingly a clear distinction of the hosts cannot be achieved using only structural data.

The GNM was computed for each protein in both the model and crystal datasets. The data generated by this coarse-grained protein dynamic model can be used to obtain information about the protein mobility at the amino acid scale. The analysis of the crystal structures revealed a certain trend in the mobility of the amino acids involved in the substrate recognition (Supplementary Figure S5). The receptor-binding site amino acids tend to be more mobile than the residues located in their proximity. A statistically significant difference between the mobilities was observed (two-sample Kolmogorov–Smirnov test equal 0.98 and *P*-value 4.89e-22). This can be partially explained by the specific role of these amino acids. Upon the binding event the receptor binding site may change conformation to accommodate the ligand in contrast to neighboring residues. Despite this trend, it should be noticed that the results for specific HA could significantly vary from one to another. This fact should be taken into account when analyzing the binding of HA and its ligand.

### Web interface

Protein distances matrices are computed using several metrics as described in the Method section. The matrices are then used by the visualization function, which represents the protein relations. Three different distance metrics can be applied to visually represent the interactions between proteins: sequence similarity score, RMSD value and ESP distance.

The web interface allows an easy interaction with the data, offering two main functionalities to the user. First the protein ‘Search’ window permits the text-based selection of proteins of interest. The service allows searching entries by id, serotype, infected host, general location, continent, year of infection, strain name and structure source (models or crystallographic structures). The Identified entries can also be alphabetically sorted by clicking at the name of a given column. For instance the expression ‘human Europe 2009’ will select HAs present in human IVA which were causing infections in Europe during the year 2009 (as shown in the Supplementary Figure S2). Moreover, by clicking at the id of a give entry, the user will be redirected to either the RCSB or to the NCBI GenBank webpage. For each entry identified with a search, the sequence, structure and electrostatic potential grid can be downloaded using the links provided in the ‘Download’ column. For each PDB entry the link to the corresponding publication is provided under the ‘Publication’ column.

**Figure 1.**
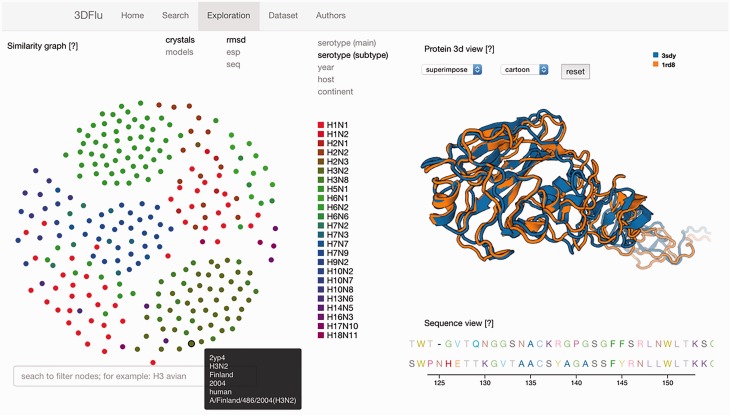
The exploration window of the database web visualization. The window includes several panels: on the left the network representation of the protein distances (ESP, RMSD, sequence similarity) with the search field; on the right the structure visualization (top) and the sequence (bottom) of the user-selected entries.

The second functionality is embedded in the ‘Exploration’ window consisting of three panels (Figure 1). The network representation panel provides the means to explore the protein relations. The user may define the data source (either crystallographic structures or homology models) and the protein metric used to visualize the interactions. All these properties can be selected using the appropriate fields located on the upper left of the panel. The color scheme applied to the network’s nodes is based on the metadata information. The user chooses a metadata category by clicking on the fields positioned on the upper right side of the panel. A specific legend is presented, depending on the selected field. The elastic network allows selecting and dragging any node. Upon changing the position of the node, the network dynamically re-adjusts the placement of all the other nodes. It is possible to highlight all the nodes sharing a common property by passing the cursor over a specific marker within the legend. Furthermore, all the available metadata information is shown when passing a cursor over a node in the dynamic network. The search bar positioned under this panel facilitates the identification of the specific network elements. The nodes with metadata matching the inserted text are then highlighted. The automatic intersection between this search and the already mentioned color scheme for the metadata category allows to easily visualize different layers of metadata information for the selected proteins. The search engine behaves in the same manner as in the ‘Search’ window

When a node is selected, the structure of the associated protein is visualized in the structural panel. The user can choose between the visualization of a single structure at a time, or the superposition of multiple structures. This feature is controlled by selecting the appropriate field from the drop down menu positioned on the top of the panel. Up to five different nodes can be selected and visualized simultaneously. Different representations (cartoon, lines, tube etc.) are available for the structures. The aligned sequences of the selected structures are displayed in the sequence panel located below the structural panel. Each amino acid type has a distinctive color to improve the visibility of the differences between the analysed the proteins. All the above mentioned functionalities were implemented using open source technologies (D3.js and PV - WebGL protein viewer). The web interface was tested on the following web browsers: Internet Explorer 11.0.29, Mozilla Firefox 45.0.1, Safari 9.1, Google Chrome 49.0.

In the ‘Dataset’ window a link to download the raw files constituting this database is provided. Thus, users can perform other structure-based analysis on the gathered data. In this window, we also provide a brief description of the data and the file organization of the database.

## Supplementary data

Supplementary data are available at *Database* Online.

## Funding

This work has been supported by the Polish National Science Centre, grant numbers (2014/15/B/ST6/05082, 2013/09/B/NZ2/00121]) and by the project Human Capital Operational Programme project ‘Information technologies: Research and their interdisciplinary applications’.

*Conflict of interest:* None declared.
